# Amyloid β oligomer induces cerebral vasculopathy via pericyte-mediated endothelial dysfunction

**DOI:** 10.1186/s13195-024-01423-w

**Published:** 2024-03-12

**Authors:** Siqi Chen, Daji Guo, Yuanyuan Zhu, Songhua Xiao, Jiatian Xie, Zhan Zhang, Yu Hu, Jialin Huang, Xueying Ma, Zhiyuan Ning, Lin Cao, Jinping Cheng, Yamei Tang

**Affiliations:** 1 Department of Anesthesiology, Sun Yat-Sen Memorial Hospital, Sun Yat-Sen University, Guangzhou, 510120 China; 2 Department of Neurology, Sun Yat-Sen Memorial Hospital, Sun Yat-Sen University, Guangzhou, 510120 China; 3 Brain Research Center, Sun Yat-sen Memorial Hospital, Sun Yat‑sen University, Guangzhou, 510120 China; 4 Nanhai Translational Innovation Center of Precision Immunology, Sun Yat-sen Memorial Hospital, Foshan, 528200 China

**Keywords:** Alzheimer’s disease, Aβ oligomer, Blood–brain barrier (BBB), Pericytes, Tight junction proteins

## Abstract

**Background:**

Although abnormal accumulation of amyloid beta (Aβ) protein is thought to be the main cause of Alzheimer’s disease (AD), emerging evidence suggests a pivotal vascular contribution to AD. Aberrant amyloid β induces neurovascular dysfunction, leading to changes in the morphology and function of the microvasculature. However, little is known about the underlying mechanisms between Aβ deposition and vascular injuries. Recent studies have revealed that pericytes play a substantial role in the vasculopathy of AD. Additional research is imperative to attain a more comprehensive understanding.

**Methods:**

Two-photon microscopy and laser speckle imaging were used to examine cerebrovascular dysfunction. Aβ oligomer stereotactic injection model was established to explain the relationship between Aβ and vasculopathy. Immunofluorescence staining, western blot, and real-time PCR were applied to detect the morphological and molecular alternations of pericytes. Primary cultured pericytes and bEnd.3 cells were employed to explore the underlying mechanisms.

**Results:**

Vasculopathy including BBB damage, hypoperfusion, and low vessel density were found in the cortex of 8 to 10-month-old 5xFAD mice. A similar phenomenon accompanied by pericyte degeneration appeared in an Aβ-injected model, suggesting a direct relationship between Aβ and vascular dysfunction. Pericytes showed impaired features including low PDGFRβ expression and increased pro-inflammatory chemokines secretion under the administration of Aβ in vitro, of which supernatant cultured with bEND.3 cells led to significant endothelial dysfunction characterized by TJ protein deficiency.

**Conclusions:**

Our results provide new insights into the pathogenic mechanism underlying Aβ-induced vasculopathy. Targeting pericyte therapies are promising to ameliorate vascular dysfunction in AD.

**Supplementary Information:**

The online version contains supplementary material available at 10.1186/s13195-024-01423-w.

## Background

Alzheimer’s disease (AD) is a fatal neurodegenerative disease characterized by senile plaques, neurofibrillary tangles, and neuronal pathology [1]. Senile plaque, as a core pathology of AD, is composed of the β-amyloid peptide (Aβ) and abnormal tau protein [2]. Therein, amyloid-beta protein, especially Aβ_42_, is reported to be the key protein that led to the development of AD. Recent reports have proved that Aβ plays an important role in AD pathology, resulting in glial activation and proliferation, neuron apoptosis, and cerebrovascular damage [3]. Most clinical treatments targeted glial and neuronal impairment in AD, such as cholinesterase inhibitors, memantine and some anti-neuroinflammatory drugs [4–6], could only enhance cognitive symptoms for a limited time period and are unable to reverse the disease course. Moreover, some of these treatments have been proven invalid, which implies an additional pathogenic mechanism of Aβ protein is still yet to be discovered [7, 8].

Increasing evidence suggests a vital role for vascular disturbance in the onset of AD. Vascular compromise including hypoperfusion and blood–brain barrier (BBB) leakage occurs early in AD [9, 10]. Exogenous amyloid β deteriorates vascular function via amplifying neuroinflammation, activating glial cells, and disturbing endothelial metabolism [11]. On the other hand, when the structure of BBB is destroyed, the transport and degradation of Aβ decline, thus enhancing Aβ abnormal accumulation and forming a negative feedback loop [12, 13]. Taken together, understanding the generation mechanism of cerebral vasculopathy may open up a brand-new sight in the development and treatment of AD [14].

When it comes to vascular impairments, many previous studies have focused on endothelial cells [15, 16]. However, pericytes and pericyte-endothelial interactions also have critical roles in maintaining normal vascular function [17]. Pericyte, as a kind of mural cell in the brain, especially locates in the small vessel (arteriole, capillary, and venule) and occupies almost 80% coverage of the abluminal side of endothelial cells [18]. As the intermediary between the blood vessels and brain parenchyma, pericytes maintain the blood–brain barrier by regulating endothelial activity such as tight junction formation and transcytosis, and guiding the astrocytic end feet polarization [19]. Pericyte controls the cerebral blood flow to coordinate neurovascular coupling as it may have the ability to contract and dilate under the stimulation of neurons [20, 21]. Besides its role in vascular function, pericyte is also found to be influential on neuroinflammatory response and metabolism [22, 23]. Regarding its vital impact on various physical activities in the brain, pericyte is believed to be relevant to many kinds of neurodegenerative diseases [24]. Unfortunately, there is limited knowledge about whether and how pericyte and pericyte-endothelial interactions are disturbed in amyloid pathology.

In this study, we explored vascular dysfunction in a transgenic AD mouse model. BBB leakage and cerebral hypoperfusion, along with pericytes and PDGFRβ decline were observed in the brains of AD mice. Further, we established a stereotaxic injection model to exclude the interference of other factors besides Aβ protein. Similar vasculopathy was found in our stereotaxic injection model. The influence of Aβ seen in vivo was analyzed mechanistically in cultured pericytes and endothelial cells.

## Methods

### Animals

Adult male wild type, 5xFAD mice, NG2-DsRed (Tg(Cspg4DsRed.T1)1Akik/J) mice and PDGFRβ-Cre; Ai9 (R26^LSL−tdTomato^) mice were obtained from the Jackson Laboratory. All the mice were of C57BL/6 background and housed in a specific-pathogen-free environment with food and water ad libitum. All animal studies were approved by the Sun Yat-sen University Animal Program Animal Care and Use Committee.

### Preparation of Aβ oligomers and stereotactic injection

The preparation of Aβ oligomer was based on previous studies [25, 26]. Briefly, synthetic Aβ1-42 (GenScript) was suspended in 1,1,1,3,3,3 hexafluoro-2-propanol (HFIP, Macklin, H811027) at 1 mM and the solution was divided into microcentrifuge tubes. Then the HFIP was removed by a Speed-Vac to obtain the Aβ peptide films for storage. Before use, the peptide films were resuspended in DMSO at 5 mM and diluted to 100 μM with PBS or DMEM/F12. Afterward, the mix was incubated at 4 °C for 24 h to generate oligomeric Aβ. For stereotactic injection, mice ranging from 2 to 3 months were anesthetized and secured in a stereotaxic frame. After that, Mice were injected with 1 μl of Aβ (100 μM) in the cortex (bregma: -1.35 mm, lateral: + 2.00 mm, depth: -0.60 mm) using a 1701RN NEUROS SYRINGE (Hamilton) at a rate of 0.1 μl/min. The region within 1 mm from the injection point was defined as the Aβo injection region and the area 1-4 mm away from the injection point as para-injection region in the ipsilateral cortex area.

### Immunofluorescence staining

After perfused with ice-cold PBS, the brain tissues of mice were harvested and fixed with 4% paraformaldehyde (PFA) at 4 °C overnight. Then 20%-30% sucrose solution was used for gradient dehydration until the brains sank to the bottom. Coronal Sects. (20 μm thick) were prepared by a freezing microtome (Leica, CryoStar NX50). After being washed with PBS three times, these brain slices were permeabilized with 0.1% Triton X-100 (Sigma) and blocked with 5% BSA (Sigma, A1933). After that, the slices were incubated with primary antibodies including mouse-anti-β-Amyloid (BioLegend, 803,004) and rabbit-anti-PDGFRβ (Abcam, ab32570) at 4 °C overnight. Appropriate secondary antibodies (1:1000, Jackson, USA), 4,6-diamidino-2-phenylindole (DAPI, 1:1000, CST, 4083S, USA) and Lectin with DyLight™ 649 Lycopersicon Esculentum (Tomato) (Vector, DL-1178–1) were used for further staining. Images were acquired with a fluorescence microscope (Leica DM6B, Germany).

### TUNEL staining

Apoptotic cells were detected by terminal deoxynucleotidyl transferase dUTP nick end labeling (TUNEL) assay (Promega, G3250) according to our previous study [27]. Briefly, the brain slices and cultured cells were blocked with 5% BSA and permeated in protease K solution at a concentration of 20 g/ml for 20 min. After incubating with the TDT reaction cocktail at 37 °C in the dark for 1 h, DAPI was stained to label the nucleus.

### Western blotting

The hippocampus and cortex of mice were extracted and lysed by RIPA buffer (Beyotime, P0013B, China) containing 1X halt™ protease and phosphatase inhibitor single-use cocktail (Invitrogen, 78,443). Afterward, Pierce™ BCA Protein Assay Kit (Invitrogen, 23,225) was used for quantitation. Samples were separated on SDS-PAGE gel and transferred to PVDF membranes (Millipore). Then we blocked the membranes with 5% milk for one hour at room temperature and incubated with primary antibodies. The primary antibodies we used include mouse-anti-β-Tubulin (1:5000, Proteintech, 66,240–1-Ig), rabbit-anti-PDGFRβ (1:1000, Abcam, ab32570), rabbit-anti-ZO1 (1:1000, ThermoFisher, 61–7300), rabbit-anti-Occludin (1:1000, ThermoFisher, 71–1500), rabbit-anti-Claudin-5 (1:1000, Abcam, ab131259), mouse-anti-tau5(1:5000, Invitrogen, AHB0042), and Phospho-Tau Family Antibody Sampler Kit (CST, 96628 T). Specific secondary HRP-linked antibodies (1:2000, Cell Signaling Technology, 7074S or 7076S) were subsequently incubated for an hour at room temperature.

### Quantitative PCR

The tissues and cells were lysed with trizol reagent (Invitrogen, 15,596,018). After extraction, PrimeScript RT MasterMix (TaKaRa, RR036A) was used to turn RNA into cDNA. QPCR was performed on a CFX96 Touch Real-Time PCR Detection System. All primers used in this study are shown in Supplementary Table 1.

### Two-photon imaging

The procedure of two-photon imaging was described in previous studies [28]. Briefly, anesthetized mice were fixed on a custom-made plate. Then a piece of skull, which is about four millimeters in diameter above the right somatosensory cortex, was removed for better imaging. 4kD and 70kD Dextran dye (Sigma-Aldrich) was injected via suborbital venous plexus. 2 to 3 fields were imaged per mouse using an Olympus 2-photon imaging system (Olympus SW FV31S, Japan).

### Laser speckle imaging

According to our previous study [29], after anesthesia with isoflurane, mice were placed on a heating pad to keep warm under the probe. Then we exposed the skull and removed the hair to acquire a clear vision. The situation of perfusion was measured by PeriCam PSI blood flow imaging software (Perimed AB, Jarfalla, Sweden).

### Cell culture and treatment

Murine pericytes were isolated as previously reported [30]. Briefly, the cortex of 3–4 weeks wild type C57BL/6 J mice were extracted and digested for half an hour by Collagenase II (Solarbio, C8150) to isolate microvessels. The cells were then cultured in a medium containing Dulbecco’s Modified Eagle Medium (DMEM, GIBCO, C11965500BT), 10% fetal bovine serum (GIBCO, 10099141C), 1% N2-supplement (GIBCO, 17,502,048), mouse recombinant EGF (Sino Biological, 50,482-MNAY), recombinant murine FGF-basic (PeproTech, 450–33-10).

### Statistical analysis

Statistical chart were produced by Prism 9.0 (GraphPad Software, San Diego, CA) and presented as mean ± SEM. Unpaired two-tailed Student’s t-test was used for two-group comparison and one-way ANOVA was performed for multiple comparisons analysis using SPSS software (IBM). Vascular analysis was accessed by Angiotool software [31]. Statistical significance was taken as *(*p* < 0.05), ** (*p* < 0.01), *** (*p* < 0.001) and ****(* p* < 0.0001).

## Results

### Vasculopathy was observed in the brains of 8-10mo 5xFAD mice

As a commonly used transgenic AD mouse model, 5xFAD mice were proved to have cognitive decline and Aβ plaque deposition at the age of 8–10 months [32]. To access the vascular change in 8-10mo 5xFAD mice, we first used two-photon imaging and laser speckle imaging technologies to visualize the structure and perfusion of cerebral vessels.

**Fig. 1:**
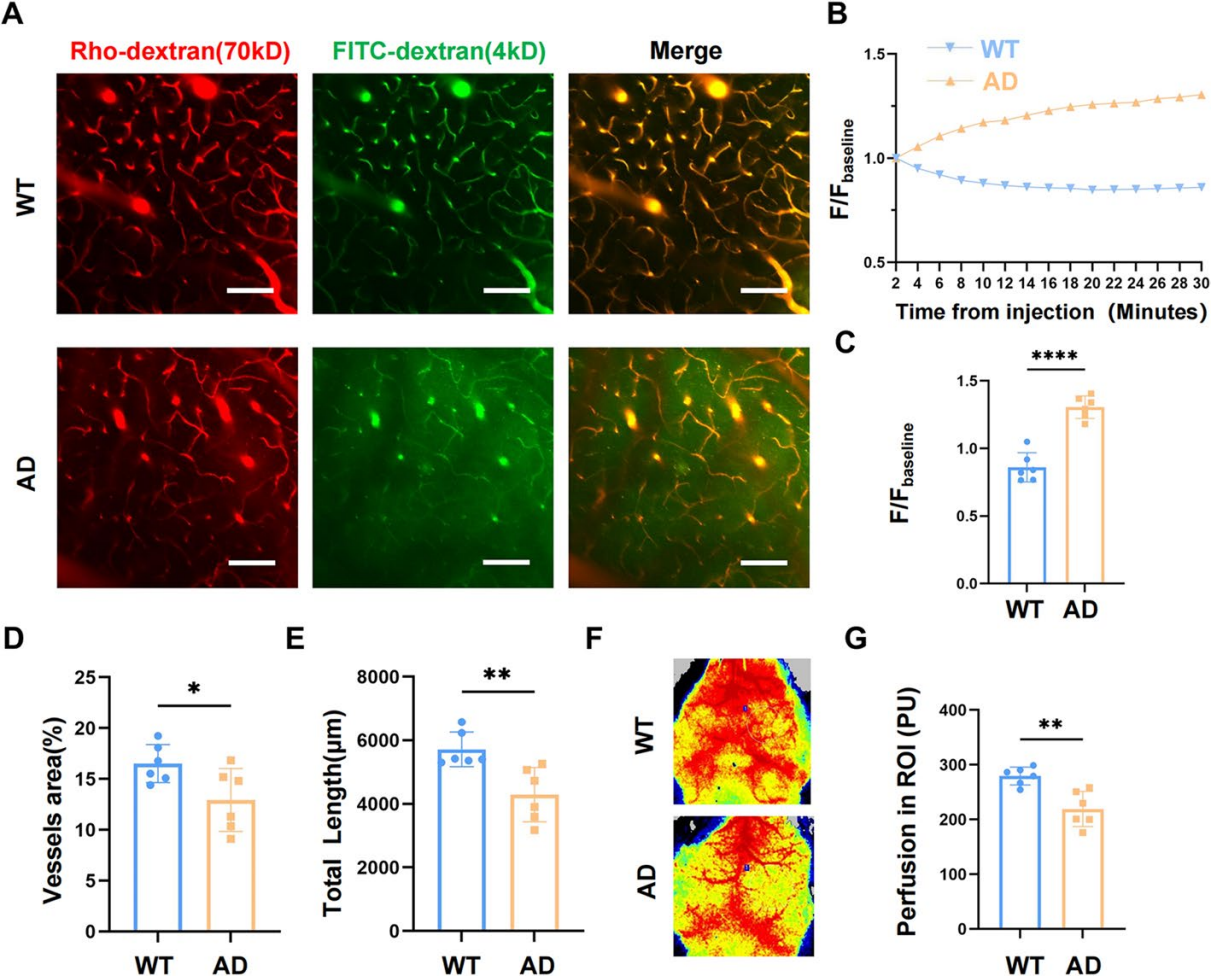
Vasculopathy was observed in the brains of 8-10mo 5xFAD mice. **A** Representative two-photon images showed cerebral vasculature and BBB breakdown in the somatosensory cortex of 8-10mo 5xFAD mice. 70kD Rhodamine-dextran was shown in red and 4kD FITC-dextran dye was in green. Scale bar: 100 μm **B**. Variation curve of the total extravascular 4kD FITC-dextran signal intensity after retro-orbital injection. **C** Volumetric quantification of FITC-dextran extravasation 30 min after injection. **D-E** Quantitative analysis of total vessel coverage area and length in the cortex of both groups. **F** Representative laser speckle images demonstrated the situation of cerebral perfusion. **G** Quantification of cerebral perfusion in ROI. All the data were presented as mean ± SEM and analyzed using unpaired t test. *n* = 6 for each group. * p < 0.05, ** p < 0.01, *** p < 0.001 and **** p < 0.0001

70kD Rhodamine-dextran was injected retro-orbitally into the 5xFAD mice and their cagemate wild type (WT) mice to display the vascular morphology and the 4kD FITC-dextran was used to evaluate the BBB permeability. When BBB broke down, intravascular 4kD FITC-dextran would leak into cerebral parenchyma while 70kD Rahodamine-dextran remained in the vascular lumen. We observed that for 5xFAD mice, the fluorescent signal of 4kD FITC-dextran dye was immediately detected in the perivascular region of the brain parenchyma and kept increasing with the extension of imaging time, suggesting a continuous BBB leakage in 5xFAD mice. Conversely, rare FITC-dextran dye was found in WT mice (Fig. 1A). Data showed the concentration of dye in the perivascular region of AD mice was about 1.5 times that of the WT mice 30 min after injection (Fig. 1B, C). Further analysis illustrated that the total vessel length (*p* < 0.01) and coverage area (*p* < 0.05) (Fig. 1D, E) were decreased in the cortex of 5xFAD mice. Laser speckle imaging showed that compared with WT mice, cerebral perfusion of 8-10mo 5xFAD mice significantly decreased (Fig. 1F, G) (*p* < 0.01), which was consistent with the loss of vessels density.

All these findings provided evidence that 8-10mo 5xFAD mice have remarkable vasculopathy, including BBB disruption, hypoperfusion and low vessel density.

### Vascular PDGFRβ deficiency was found in the brains of 8-10mo 5xFAD mice

**Fig. 2:**
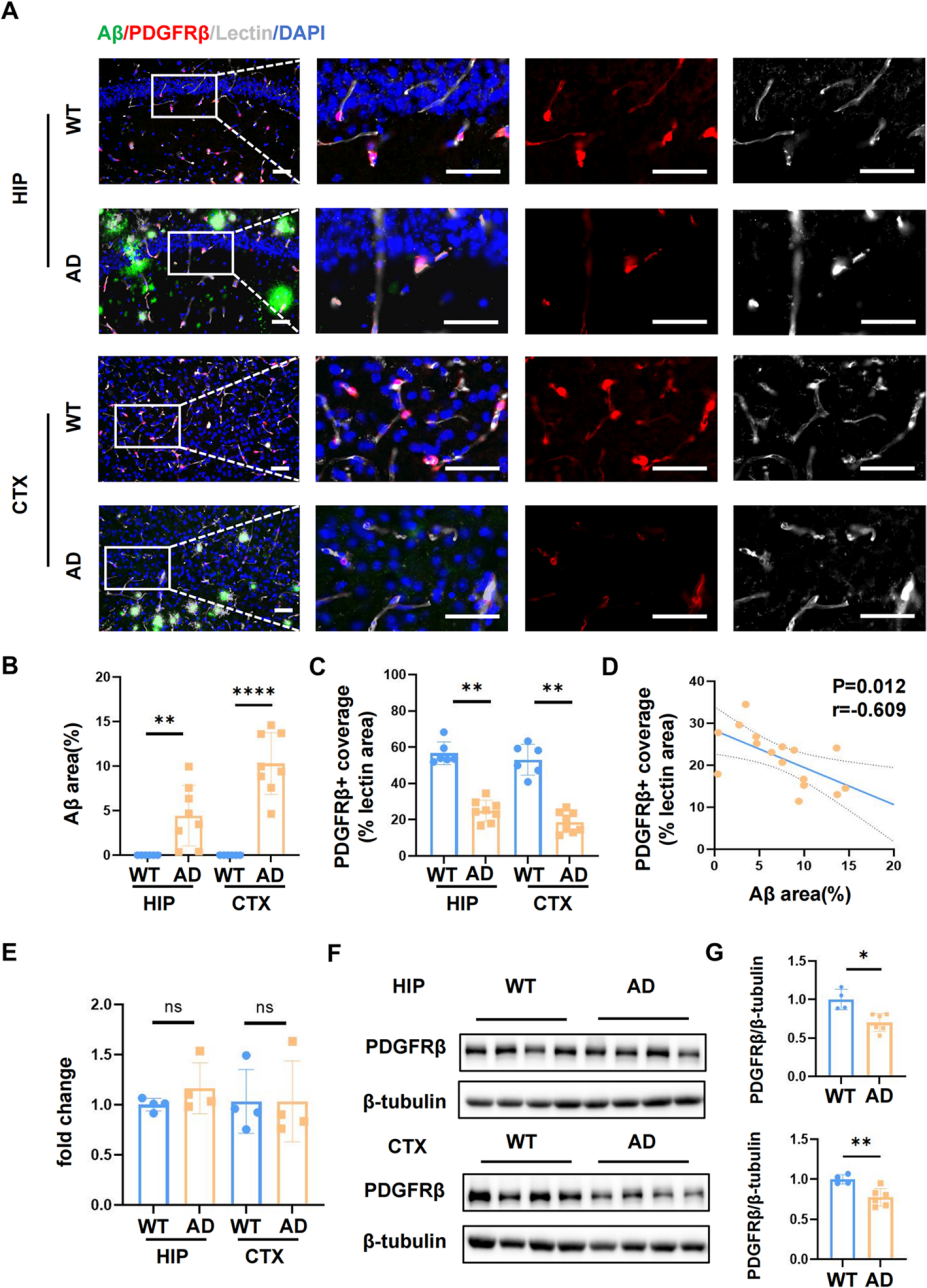
Vascular PDGFRβ deficiency was found in the brains of 8-10mo 5xFAD mice. **A** Representative immunofluorescent staining images of PDGFRβ (red), Aβ (green) and lectin (grey) co-labeling in the hippocampus (upper panel) and cortex (low panel) of 8-10mo wild type (*n* = 6) and 5xFAD (*n* = 8) mice. Scale bar: 50 μm **B**. Quantification of Aβ-positive area in both groups. **C** Quantification of PDGFRβ-positive coverage area. **D** Pearson’s coefficient (r) correlation analysis between the percentage of PDGFRβ^+^ area and Aβ.^+^ area in the cortex of AD mice. **E** Quantitative analysis of PDGFRβ mRNA levels. *n* = 4 for each group. **F-G** Western blotting analysis of PDGFRβ protein expression level in the hippocampus (upper panel) and cortex (low panel) of wild type (*n* = 4) and 5xFAD mice (*n* = 6). Data are shown as mean ± SEM. * p < 0.05, ** p < 0.01, *** p < 0.001, and **** p < 0.0001

Since pericytes are important for BBB integrity and capillary constriction [33, 34], we next explored the alteration of pericytes in 5xFAD mice. According to previous studies, PDGFRβ, which was mainly expressed in capillary pericytes in the brain, was used as the marker of pericytes [35]. Immunofluorescence staining results demonstrated an evident Aβ protein deposition alongside the loss of PDGFRβ signal in the hippocampus CA1 region and cortex of the AD mice model (Fig. 2A, B). Although the number of PDGFRβ^+^ cells remained constant, the percentage of PDGFRβ^+^ area covering lectin-labeled vessels dramatically declined no matter in the hippocampus (*p* < 0.01) or the cortex (*p* < 0.01) of 8-10mo 5xFAD mice (Fig. 2C).

We further confirmed the mRNA and protein level of PDGFRβ by qPCR and Western blotting respectively. Data revealed that the mRNA level of PDGFRβ stayed the same (Fig. 2E), while the protein level of PDGFRβ decreased in the CA1 region (*p* < 0.05) and cortex (*p* < 0.01) of 5xFAD mice compared to WT controls (Fig. 2F, G). Surprisingly, the PDGFRβ protein level was cut down more obviously in the cortex region. Interestingly, Pearson’s correlation analysis implied that PDGFRβ loss was possibly associated with Aβ deposition area in the cortex of 5xFAD mice (*p* < 0.05). This suggested that vascular PDGFRβ deficiency in AD might be attributed to the vasotoxic effects of Aβ protein.

### Aβ oligomer injection induced BBB leakage and local cortex hypoperfusion in 2mo WT mice

**Fig. 3:**
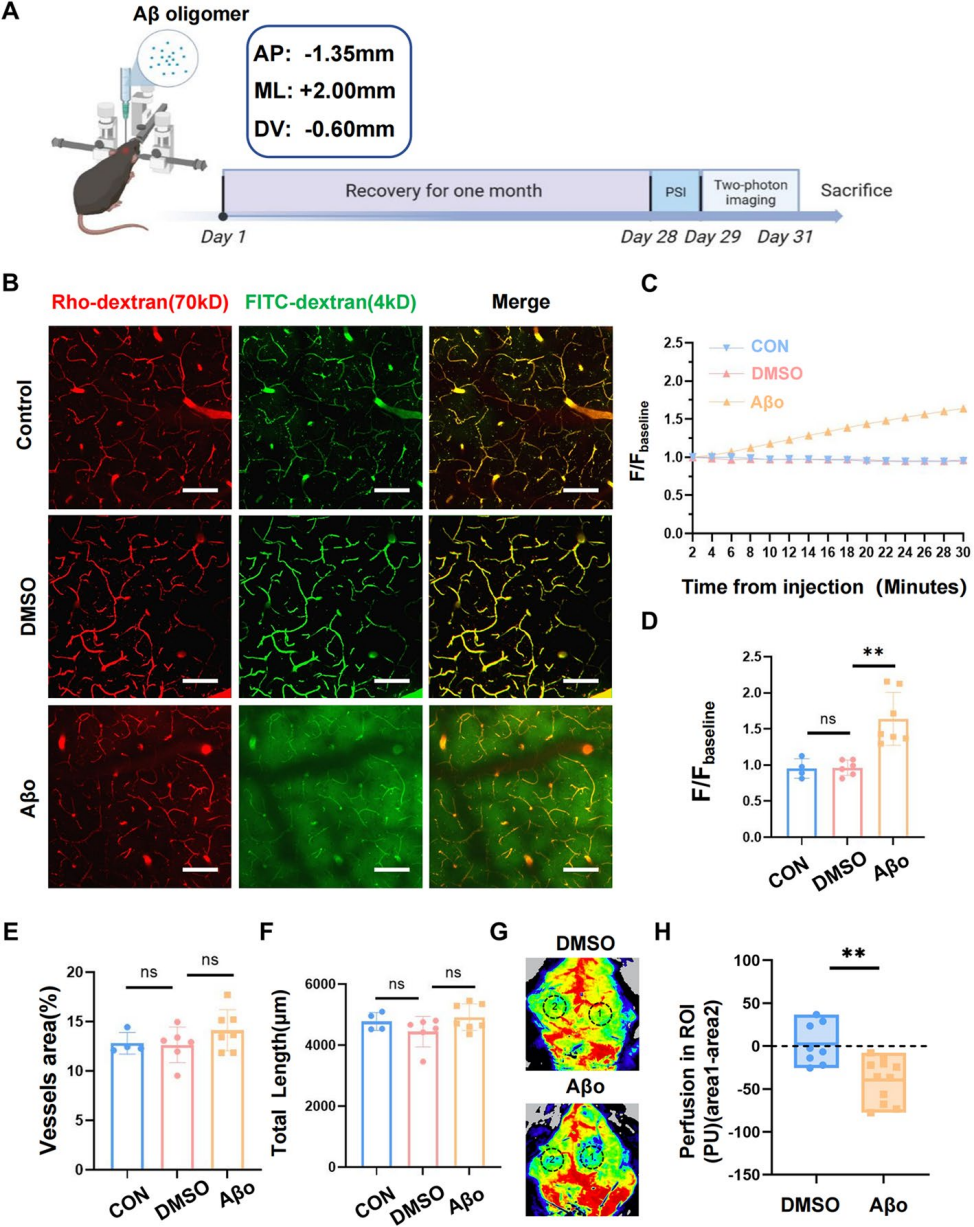
Aβ oligomer stereotactic injection induced BBB leakage and local cortex hypoperfusion in 2mo WT mice. **A** Schematic diagram of the experiment design. **B** Representative in vivo two-photon images of the cortex of mice in control (*n* = 4), vehicle (2%DMSO) (*n* = 6), and Aβo-injected (*n* = 7) groups. 2–3 stacks were acquired to obtain the mean for each animal. Scale bar: 100 μm **C**. Variation curve of extravasated FITC-dextran dye intensity in these three groups after injection. **D** Quantitative analysis of FITC-dextran extravasation 30 min after injection. **E–F** Quantification of cortical vascular length and area from the three experimental groups. **G** Representative laser speckle images of DMSO (*n* = 8) and Aβo (*n* = 11) groups. **H** Quantification of D-value of cerebral perfusion between injection region (ROI 1) and contralateral cortex (ROI 2) in DMSO and Aβo groups. D-value was defined as the ROI 1 quantitative perfusion value minus ROI 2. All the data were presented as mean ± SEM. * p < 0.05, ** p < 0.01, and *** p < 0.001

To further characterize the role of Aβ protein in cerebrovascular dysfunction and eliminate the effects of other pathogenic factors in AD, we established a stereotactic injection model (Fig. 3A, Supplementary Fig. 1A). First, soluble Aβ42 protein was processed into oligomer for Aβ oligomer (Aβo) was proved to be more toxic than Aβ monomer and fiber in both neuronal [36–38] and vascular level [39, 40]. Dot blotting was used to confirm that Aβo was produced successfully (Supplementary Fig. 1B). Then we injected Aβo into the cortex of two-month-old mice to mimic Aβ pathology in AD and further confirmed the success of injection by immunofluorescence staining immediately after the operation (Supplementary Fig. 1C). To rule out the influence of stereotactic injection, we injected the solvent of Aβo (2%DMSO) as a better control group.

Two-photon imaging revealed that the solvent administration group did not exist BBB leakage after recovering from injection for a month. There was almost no difference between the DMSO group and the control group without any treatment. On the contrary, the Aβo administration group showed remarkably increased BBB permeability (*p* < 0.01) (Fig. 3B-D). Interestingly, we found that the total vessel area and length did not change (Fig. 3E, F), suggesting that the decreased capillary density might be a delayed symptom in AD and might appear after a longer Aβo treatment time. We, therefore, focused on the perfusion situation in this stereotactic injection model. It is worth noting that compared to the contralateral side, Aβo injection induced local cortex hypoperfusion around the injection point (Fig. 3G, H) (*p* < 0.01). Previous studies have demonstrated that Aβ has great influence in tau oligomerization, aggregation and phosphorylation [41]. Additionally, there is increasing evidence that the abnormal tau proteins also take part in BBB breakdown [42]. Thus, we detected the protein levels of total tau and phosphorylated tau at residues Ser202/ Thr205, Thr181, Ser396 in the cortex of Aβo-injected wild type mice, as well as in healthy controls. The results showed no significant changes in the levels of all these proteins one month after Aβ oligomer injection (Supplementary Fig. 1D, E). These findings indicated that in our study, a single stereotactic injection of Aβ was insufficient to induce an increase in tau pathology, suggesting that Aβ itself may contribute to the vasculopathy seen in AD. All these results claimed that we successfully established a model to imitate vascular dysfunction in AD and proved that Aβ was able to result in vasculopathy independently.

### NG2 expression decreased after Aβo injection in NG2-DsRed mice

**Fig. 4:**
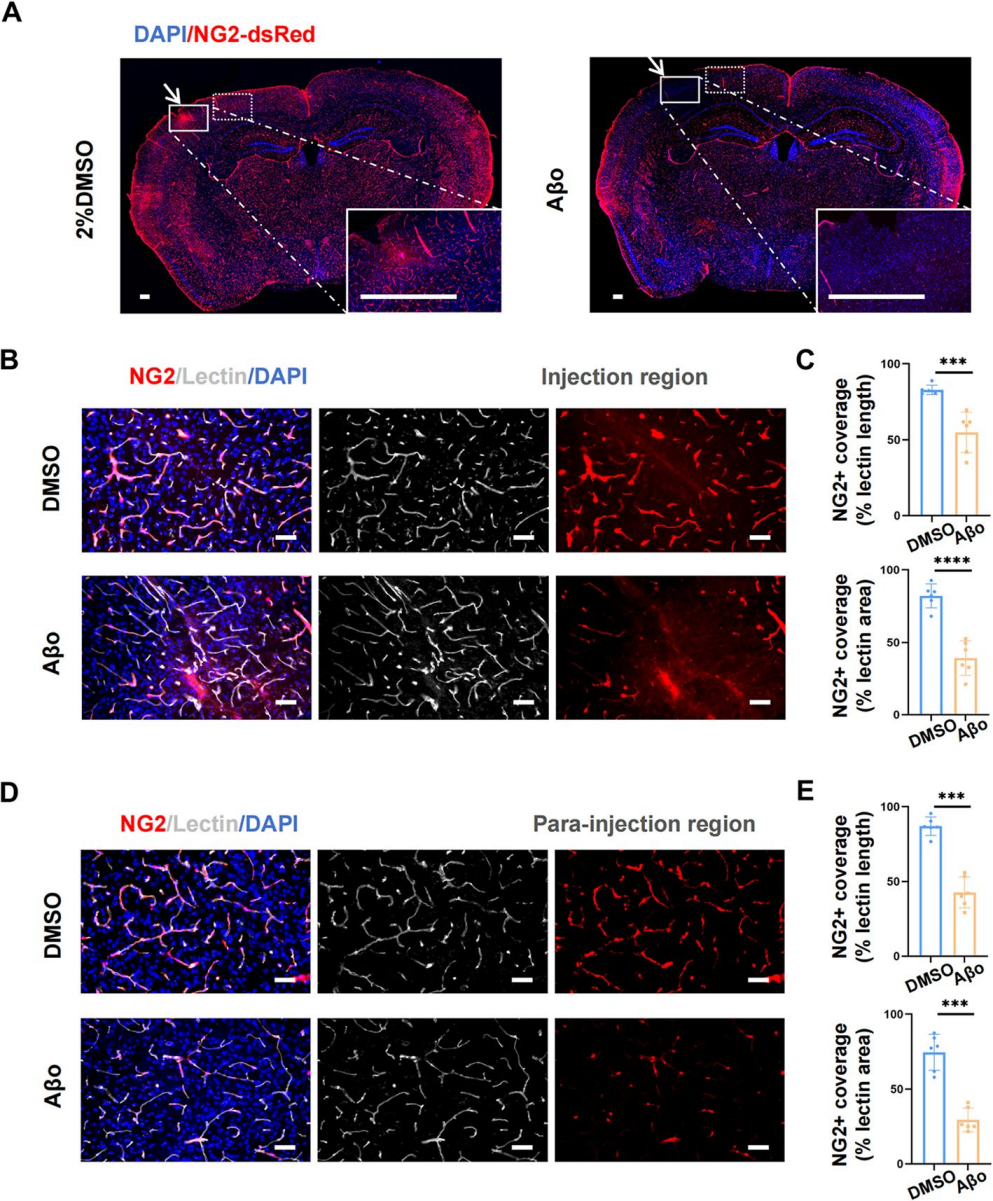
The level of NG2 expression decreased after Aβo injection in NG2-DsRed mice. **A** Representative images of coronal brain section of the vehicle (2%DMSO) and Aβo-injected NG2-DsRed mice. The area 1 mm around the injection point (the arrow points) was defined as the injection region and the ipsilateral cortex area 1-4 mm away from the injection point was defined as the para-injection region. Scale bar: 250 μm **B** Representative immunofluorescent staining images of NG2 (red) and lectin (grey) in the injection region of both groups. Scale bar: 50 μm **C** Quantification of NG2-positive coverage length (upper panel) and area (low panel) in the injection region of both groups. **D** Representative immunofluorescent staining images of NG2 (red) and lectin (grey) in the para-injection region of both groups. Scale bar: 50 μm **E** Quantification of NG2-positive coverage length (upper panel) and area (low panel) in the para-injection region of both groups. All the data were presented as mean ± SEM and analyzed using an unpaired t-test. *n* = 6 for each group. * p < 0.05, ** p < 0.01, *** p < 0.001 and **** p < 0.0001

We next used NG2-DsRed mice to examine whether there existed pericyte loss in this Aβo stereotactic injection model. Similar to PDGFRβ, NG2 is another pericyte marker [43]. We observed that the NG2-positive signal dramatically diminished in the Aβo injection region compared with the DMSO group (Fig. 4A). NG2-positive cells reduced by about 20% in the Aβo-injected region (Supplementary Fig. 2B). Furthermore, the percentage of coverage length and area of NG2-positive cells notably declined at the injection point (Fig. 4B, C) (*p* < 0.001, *p* < 0.0001). It was interesting that the length and area of NG2-positive cells also downregulated after Aβo administration in the para-injection region, which was defined as the ipsilateral cortex area 1-4 mm away from the injection point (Fig. 4D, E) (*p* < 0.001, *p* < 0.001). The number of NG2-positive cells in the para-injection region also decreased in the Aβo group (Supplementary Fig. 2B). We speculated that this phenomenon was attributed to the propagation of Aβ as indicated by previous studies [44, 45]. Tunel staining suggested that the change of NG2 was not owing to pericyte apoptosis (Supplementary Fig. 2A). These results above indicated that Aβo injection inhibited the expression of NG2 around the injection point.

### Aβo injection inhibited the expression of PDGFRβ and induced an inflammatory response in PDGFRβ-cre: Ai9 mice

**Fig. 5:**
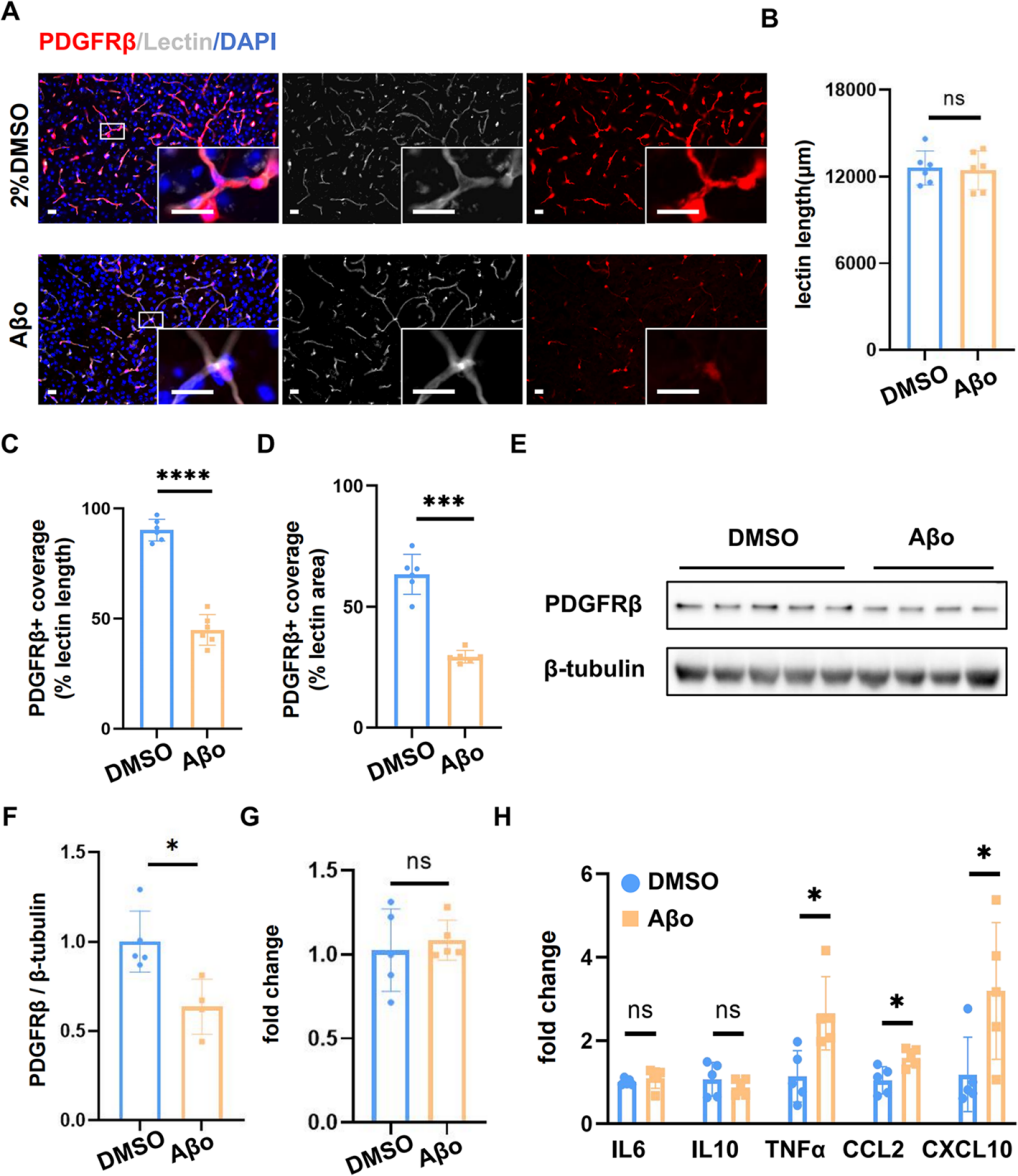
Aβo injection inhibited PDGFRβ expression and induced an inflammatory response in PDGFRβ-cre: Ai9 mice. **A** Representative immunofluorescent staining images of PDGFRβ (red) and lectin (grey) in the injection region of vehicle (2%DMSO) and Aβo-injected NG2-DsRed mice. Scale bar: 20 μm **B** Quantification of the total length of lectin^+^ area. **C-D** Quantification of PDGFRβ.^+^ coverage length (left panel) and area (right panel) in the injection region of both groups. **E–F** Western blotting analysis of PDGFRβ protein expression level in brain tissues after 2%DMSO and Aβo injection. **G** QPCR analysis of PDGFRβ mRNA expression level in brain tissues after vehicle and Aβo injection. **H** QPCR analysis of mRNA levels of some classic inflammatory factors (IL6, IL10, TNFα, CCL2, CXCL10) in brain tissues. All the data were presented as mean ± SEM and analyzed using an unpaired t-test. *n* = 6 for each group. * p < 0.05, ** p < 0.01, *** p < 0.001 and **** p < 0.0001

In consideration of their high heterogeneity, pericytes are still hard to identify in the brain today [18]. To ensure the loss of the NG2 signal was due to the pericyte degeneration, we used another transgenic mouse model, which was obtained from the PDGFRβ-cre mice crossed with Ai9 reporter mice, to label pericytes. Immunofluorescence staining detected a similar decrease in PDGFRβ-positive cells coverage in lectin^+^ length (*p* < 0.0001) and area (*p* < 0.001) in the Aβo injection region (Fig. 5A, C, D). What’s more, the number of PDGFRβ-positive cells declined significantly compared with the DMSO group. Interestingly, the total length of the lectin^+^ area shows no significant change between Aβo injection and control groups (Fig. 5B), which is correlated with the former result we observed from the two-photon microscope, indicating that administration of Aβo influenced pericytes more severely and rapidly than endothelial cells. Furthermore, we extracted the hypoperfusion brain tissue about 4 mm in diameter according to laser speckle imaging results. Western blotting revealed significant down-regulation of PDGFRβ expression (*p* < 0.05) after Aβo injection (Fig. 5E, F). Consistent with our data in AD mice, the mRNA level of PDGFRβ did not show any difference (Fig. 5G), indicating that the alternation of PDGFRβ was only limited to the protein level. Taken together, we found that pericytes underwent degeneration in our Aβo injection model.

Neuroinflammation was considered to play a key role in the progression of AD [46]. To examine the effects of Aβo in neuroinflammatory, we detected the mRNA level of some classic inflammatory cytokines in our stereotactic injection model. There is no difference in IL6 or IL10 between the DMSO and the Aβo group, while the mRNA levels of TNFα (*p* < 0.05), CCL2 (*p* < 0.05), CXCL10 (*p* < 0.05) were significantly elevated (Fig. 5H). These findings revealed that Aβo exaggerated neuroinflammation in the injection area.

### Pericytes incubated with Aβo demonstrated a proinflammatory profile and affected endothelial cells via reducing tight-junction proteins in vitro

**Fig. 6:**
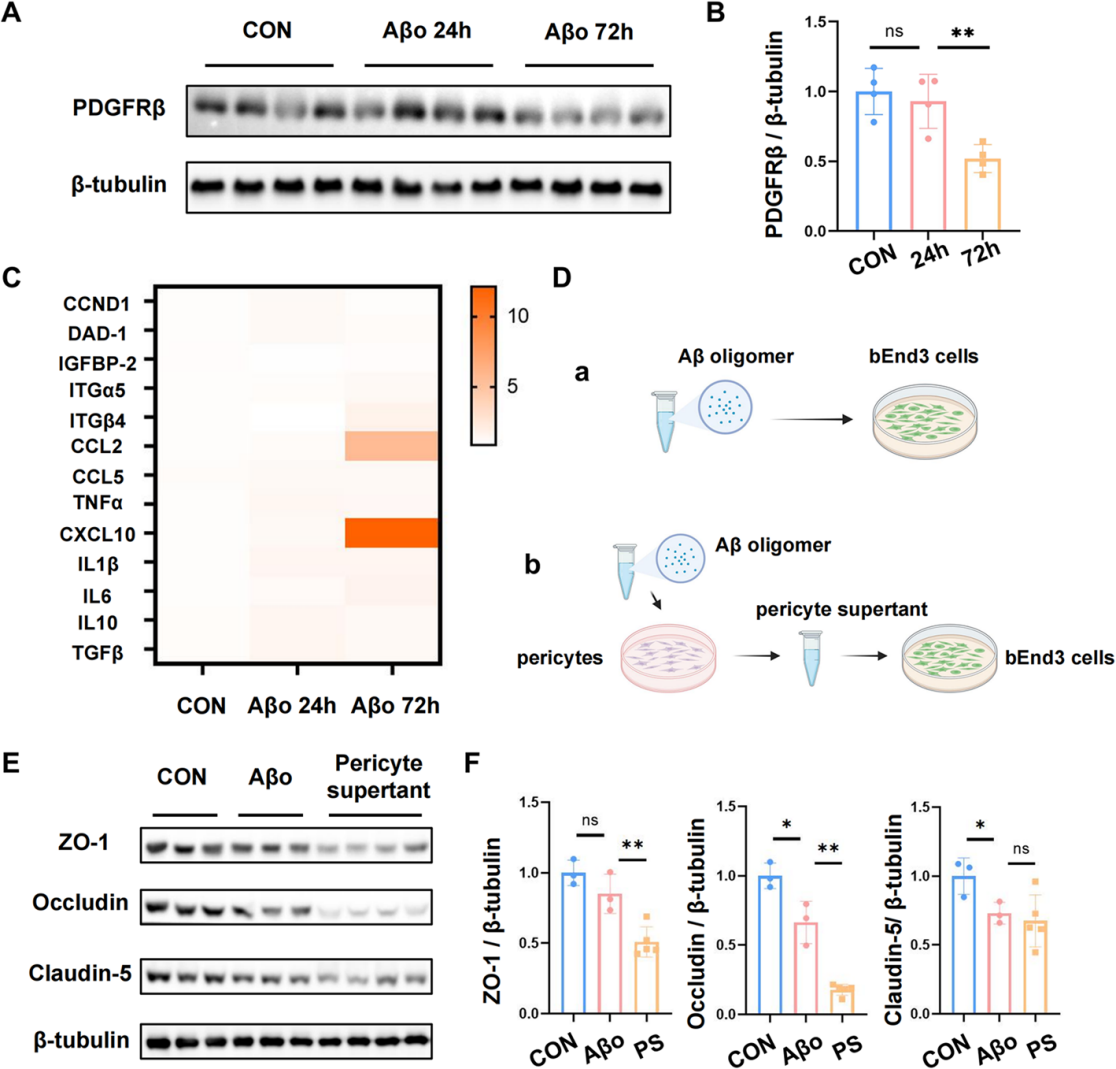
Aβo-incubated pericytes demonstrated a proinflammatory profile and decreased tight-junction proteins in endothelial cells in vitro. **A-B** Western blotting analysis of PDGFRβ protein expression level in primary cultured pericytes incubated with Aβo for 24 h and 72 h. **C** Heat map summary of Aβo-stimulated genes in primary pericytes incubated with Aβo for 24 h and 72 h. **D** Schematic diagram of bEnd3 cells incubated with primary pericytes supernatant. **E–F** Western blotting analysis of tight junction protein (ZO-1, Claudin-5, and Occludin) expression levels in bEnd3 cells incubated with Aβo and primary pericytes supernatant. All the data were presented as mean ± SEM. * p < 0.05, ** p < 0.01

To further confirm that Aβo was able to reduce the expression of PDGFRβ in pericytes, primary cultured pericytes were incubated with Aβo for 24 h and 72 h at a concentration of 10 μM in vitro. Consistent with the results above, the level of PDGFRβ slightly declined after 24 h and remarkably reduced three days after Aβo treatment (Fig. 6A, B) (*p* < 0.01). We first explored whether the decline of PDGFRβ was due to the death of pericytes. The viability of pericytes detected by the CCK-8 assay decreased proportionally with treatment time and concentration while incubating with Aβ oligomers (Supplementary Fig. 3A). Tunel staining illustrated that rare pericytes underwent apoptosis after Aβo treatment (Supplementary Fig. 3B). Next, we used qPCR to investigate possible mediators between pericytes and endothelial cells according to the comparative cDNA expression array data in a previous study [47]. We found that the mRNA levels of some traditional inflammatory cytokines, such as IL6, IL10, IL1β, TNFα, and TGFβ, demonstrated an increasing trend in pericytes incubated with Aβo for three days. Besides, some cytokines associated with cell death, including cyclin D1(CCND1) and defender against cell death-1 (DAD-1), were also elevated. Among those potential genes, the changes in chemokine family expression in pericytes were noticeably higher than the other genes. The mRNA level of CCL2 in pericytes treated with Aβo for 72 h was about four times more than that of the control group. For CXCL10, the gene expression reached approximately 10 times (Fig. 6C). These findings showed that Aβo inhibited the expression of PDGFRβ and raised the secretion of inflammatory factors in primary pericytes.

Moreover, we sought to evaluate whether the TJ proteins in bEnd.3 cells underwent degeneration after incubating with the supernatant of pericytes treated with Aβo. Given that Aβo has been implicated with the decrease of TJ proteins in endothelial cells [48], we added a group of bEnd.3 cells which were incubated with Aβo at a dose of 10 μM for better comparability (Fig. 6D). Unsurprisingly, the expression of TJ proteins, including ZO-1, Occludin (*p* < 0.05) and Claudin 5 (*p* < 0.05), was reduced after Aβo administration. Importantly, after incubating with the supernatant of Aβo-treated pericytes, bEnd.3 cells demonstrated much lower protein levels of ZO-1 (*p* < 0.01), Occludin (*p* < 0.01) and Claudin 5(Fig. 6E, F). Together, these data suggested that pericytes incubated with Aβo aggravated the injury of TJ proteins in endothelial cells.

## Discussion

Ever since George G. Glenner and Caine W. Wong discovered the Aβ protein in the brains of AD patients in 1984 [49], the downstream mechanisms of this abnormal-folded protein attracted a lot of attention internationally. To cure this fatal neurodegenerative disease, therapies targeting the Aβ protein have been hot spots for a long time. However, many drugs failed to reach the expectation of slowing down the progress of AD in clinical trials, raising doubts about the “amyloid cascade hypothesis” [50, 51]. Some researchers believe that Aβ acts more like a trigger or accelerator rather than a direct cognitive effector in AD [52]. On the contrary, they consider that the miss-folded tau protein, as another important pathological protein, has a more important influence on cognitive function due to its neuronal and synapse toxicity [53]. Recently, a successful phase 3 clinical trial of donanemab, an Aβ monoclonal antibody, restored faith in the Aβ hypothesis. Among participants at the early stage of AD, donanemab was proven to slow clinical progression at 76 weeks [54]. In addition, ACI-24, an anti-Aβ vaccine, received fast-track approval from the U.S. Food and Drug Administration (FDA) on June 27, 2023 [55]. All these successful trials reconfirm the vital role of Aβ in AD and indicate a bright future for anti-Aβ therapies. In our study, we strengthened the reliability of anti-Aβ therapies by providing new insight into its vasotoxicity in Aβ-related pathology. We revealed a relationship between Aβ pathology and cerebral vasculopathy in 5xFAD mice and an Aβ-injected mouse model, identified the associated molecular changes in pericytes and explored the underlying mechanisms.

Vascular dysfunction, including BBB leakage and reduction in cerebral blood flow, is reported to play an important role in the progress of AD [56, 57]. Vasculopathy is not exclusive to the later clinical symptoms of AD, but also happens in patients with early amyloid and tau pathology [14]. A retrospective study has found patients with cerebrovascular accidents suffer a much higher risk of AD than others [58]. According to a previous clinical study, BBB injury was considered as one of the early biomarkers of cognitive impairment in AD patients [59]. BBB leakage allows infiltration of peripheral immune cells into the brain, and accumulation of peripheral protein and poisonous substance in cerebral parenchyma, such as fibrinogen, globulin, and exogenous Aβ protein, both of which contribute to neuroinflammation and neuronal damage [60, 61]. Non-structural vascular abnormalities also took a considerable part in AD. Hemodynamic abnormalities such as hypoperfusion and microthrombosis induce regional hypoxia and neurovascular uncoupling, hence worsening the process of AD [14, 62]. In our research, we first detected BBB breakdown and hypoperfusion in 8-10mo 5xFAD mice, supporting the concept that vasculopathy indeed appeared in the process of AD. To find out the cause of BBB leakage, we used immunofluorescence staining and western blot to identify PDGFRβ deficiency in the brain capillaries of 5xFAD mice. PDGFRβ deficiency implied that there might be a pericyte loss or detachment in microvessels. This is consistent with a previous study that uncovered that pericyte implantation increased cerebral perfusion and alleviated Aβ pathology in AD model mice [63]. Angio-analysis recovered that in AD mice, the total length and area of brain vessels were decreased, which partly explained the hypoperfusion phenomenon, but whether decreased vessel density, hypoperfusion and later hypoxia are sufficient to be responsible for AD-related neuronal impairment needs further examination. Of note, the possible locational co-relation of Aβ deposits and PDGFRβ loss in 5xFAD mice gave us a hint that the relationship between pericytes and Aβ was worth to be explored.

As a composition of BBB, pericytes were of great importance in vascular homeostasis [33]. BBB-associated pericytes were able to clear Aβ deposition via an LRP1-dependent ApoE isoform-specific pathway [64]. Unfortunately, only a few studies focused on the relationship between Aβ and pericytes [65]. Previous research disclosed that Aβ oligomers evoked ROS generation in pericytes, thus increasing the release of endothelin-1 (ET-1). The increasing level of ET-1 was associated with pericyte contraction, resulting in constriction at the pericyte location in human capillaries [25, 66]. Moreover, several studies suggested that Aβ protein enhanced the level of matrix metalloproteinase-9 (MMP-9), increased the activity of caspase 3/7, decreased viability and proliferation, and even led to death in pericytes [39, 67]. Herein, we set up a model to uncover the direct relationship between Aβ protein and vascular pathology via injecting Aβo into the brain cortex. Interestingly, we found significant differences in the expression of PDGFRβ and NG2 accompanied with similar hypoperfusion and BBB breakdown in this mouse model, suggesting that pericytes indeed took an important part in Aβ-induced vasculopathy.

When it comes to the mechanisms underlying pericyte-associated BBB breakdown, we first examined whether there existed pericyte death after Aβ treatment. To our surprise, Tunel staining showed rare cell apoptosis appeared in our model. Besides, the number of pericytes decreased slightly while the coverage of pericytes significantly changed. All these dropped a hint that non-structural changes in pericytes might be the leading part of the pathogenesis. Similar to endothelial cells, pericytes also function as an important regulator of microenvironment homeostasis through secreting several factors. Pericytes were proved to be the first responder to systemic inflammation. Immediately after LPS injection, pericytes rapidly secreted chemokine CCL2 and were the major source of inflammatory factors within the first two hours [68]. Another research in the brains of cocaine abusers illustrated that pericytes, other than endothelial cells, played a vital role in expressing CXCL10, which brought about monocyte infiltration and NF-κB-related neuroinflammation [69]. According to a previous in-vitro study, human brain pericytes showed separate gene expression after incubating with Aβo [47]. We observed similar alternations in mouse primary pericytes in vitro. The expression of CCL2, CXCL10, and other inflammatory factors was significantly raised after Aβo administration. These changes increased over time and further studies are needed to verify their influence.

### Limitations of the study

While our study is a meaningful step in Aβ-induced pathology, it also has some limitations. One concern about the findings was that the number of mice used to evaluate the situation of cerebral vasculopathy and pericyte loss was kept low for feasibility and ethical purposes. What’s more, we did not put our emphasis on the hippocampus-associated changes and cognitive dysfunction because we focused on the alternation of the somatosensory cortex due to the limited detection depth of two-photon imaging technology. Lastly, more experiments were needed to explore the underlying mechanisms of the interactions between pericytes and endothelial cells.

## Conclusions

Taken together, our work identified evident vasculopathy and PDGFRβ deficiency in 8-10mo 5xFAD mice. Along with pericyte degeneration, an Aβo stereotactic injection mouse model demonstrated similar vascular dysfunction such as BBB leakage and hypoperfusion. The increased levels of inflammatory factors in pericytes after Aβo treatment inhibited the expression of TJ proteins in endothelial cells. We revealed that targeting pericyte therapies may be promising strategies for the prevention and therapy of Aβ-induced vasculopathy.

### Supplementary Information

**Additional file 1: Figure S1.** Dot blotting and immunofluorescent staining to ensure successful Aβ oligomerization and effective injection. A. Schematic diagram of the Aβo injection point B. Representative images of dot blotting. C. Representative immunofluorescent staining images of Aβ (green) and DAPI at the injection point 10 min and 30 min after Aβo treatment. Scale bar: 50 μm D-E. Western blotting analysis of total tau (tau5) and phosphorylated tau at residues Ser202/ Thr205 (AT8), Thr181, Ser396 expression levels in the injection cortex. All the data were presented as mean ± SEM.

**Additional file 2: Figure S2.** The number of pericytes declined after Aβo treatment and rare apoptosis was found in Aβo-injected NG2-DsRed mice. A. Representative images of NG2 (red), tunel (green) and DAPI (blue) in the injection region and para-injection region of vehicle (2%DMSO) and Aβo-injected NG2-DsRed mice. B. Quantification the number of NG2 + cells(/mm2) in the injection region and para-injection region of the vehicle (2%DMSO) (*n* = 6) and Aβo-injected (*n* = 6) groups. C. Quantification of the number of PDGFRβ + cells(/mm2) in the injection region of vehicle (2%DMSO) (*n* = 6) and Aβo-injected (*n* = 6) groups. All the data were presented as mean ± SEM and analyzed using an unpaired t-test. ** p < 0.01 and **** p < 0.0001

**Additional file 3: Figure S3.** The viability of primary pericytes decreased after Aβo incubation and rare apoptosis was found in vitro. A. The OD value (450 nm) of primary pericytes detected by CCK-8 assay after incubated in media containing different concentrations of Aβ oligomers (0, 5, and 10 μM) for 24 h and 72 h. B. Representative images of tunel (green) and DAPI (blue) in primary pericytes incubated with Aβo for 24 h and 72 h. Scale bar: 50 μm. All the data were presented as mean ± SEM. **** p < 0.0001

**Additional file 4: Supplementary Table 1.** List of primers used in RNA analyses.

## Data Availability

The datasets used and/or analysed during the current study are available from the corresponding author on reasonable request.

## Abbreviations

AD: Alzheimer’s disease
Aβ: Amyloid-β
BBB: Blood-brain barrier
PDGFRβ: Platelet-derived growth factor receptor-β
TJ: Tight-junction
WT: Wild type
Aβo: Amyloid beta oligomer
CCND1: Cyclin D1
DAD-1: Defender against cell death-1
MMP-9: Metalloproteinase-9
ET-1: Endothelin-1
