# Supplementary figures and images for: Amyloid β oligomer induces cerebral vasculopathy via pericyte-mediated endothelial dysfunction
Source: Alzheimers Res Ther. 2024 Mar 12;16:56. doi: 10.1186/s13195-024-01423-w (PMC10935813; doi:10.1186/s13195-024-01423-w)

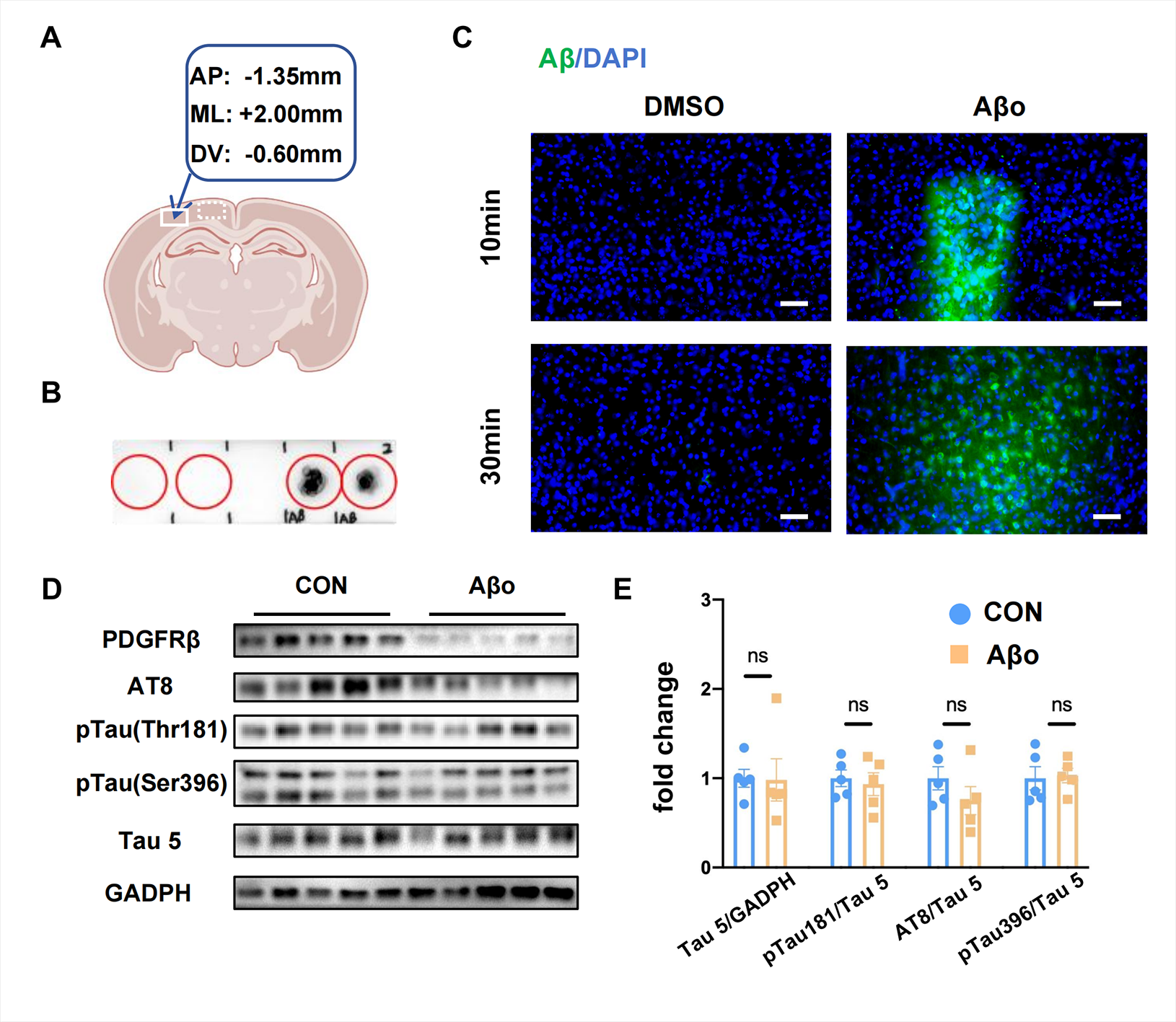

Supplement: Supplementary file 1 — Additional file 1: Figure S1. Dot blotting and immunofluorescent staining to ensure successful Aβ oligomerization and effective injection. A. Schematic diagram of the Aβo injection point B. Representative images of dot blotting. C. Representative immunofluorescent staining images of Aβ (green) and DAPI at the injection point 10 min and 30 min after Aβo treatment. Scale bar: 50 μm D-E. Western blotting analysis of total tau (tau5) and phosphorylated tau at residues Ser202/ Thr205 (AT8), Thr181, Ser396 expression levels in the injection cortex. All the data were presented as mean ± SEM. [file 13195_2024_1423_MOESM1_ESM.tif]

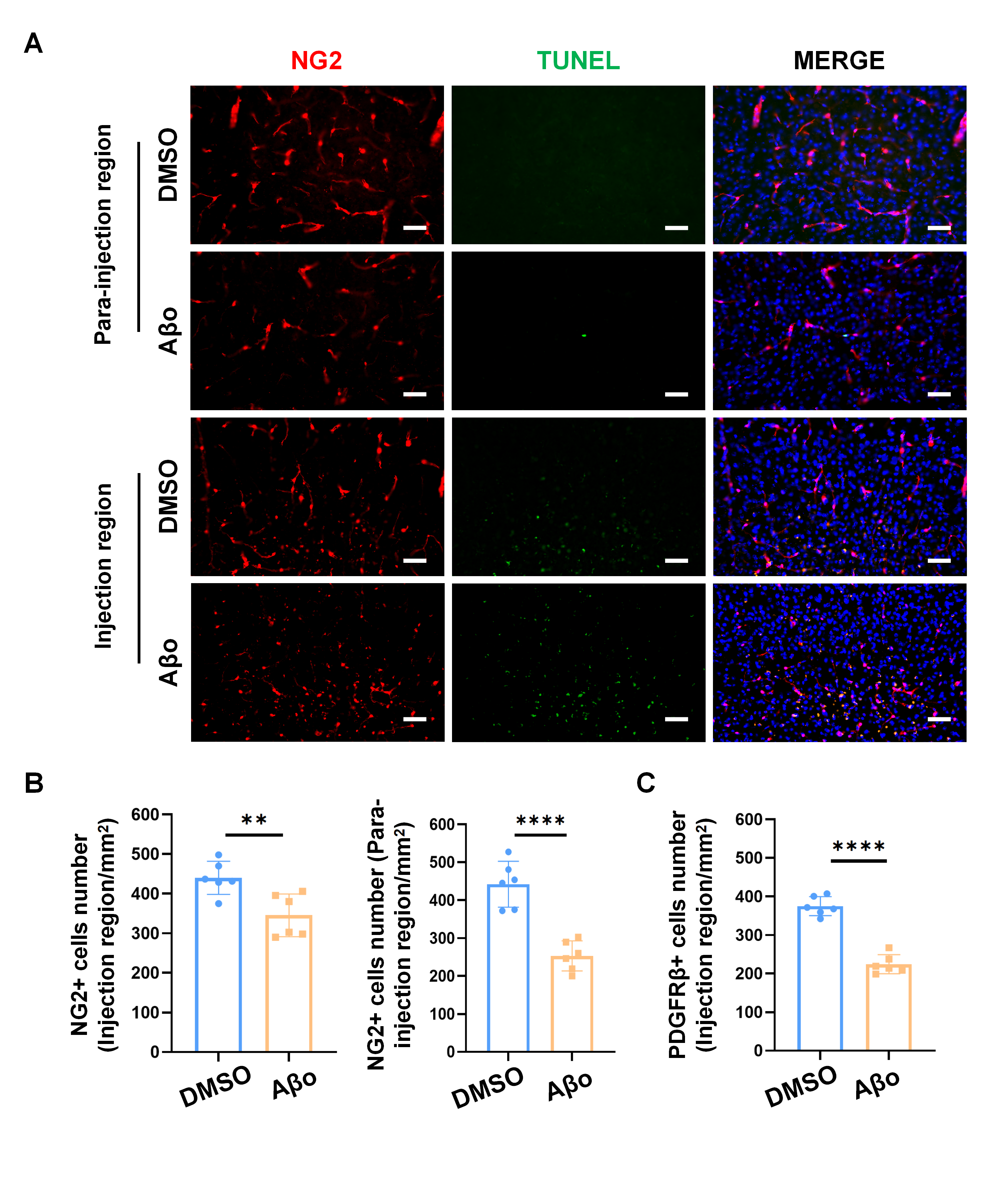

Supplement: Supplementary file 2 — Additional file 2: Figure S2. The number of pericytes declined after Aβo treatment and rare apoptosis was found in Aβo-injected NG2-DsRed mice. A. Representative images of NG2 (red), tunel (green) and DAPI (blue) in the injection region and para-injection region of vehicle (2%DMSO) and Aβo-injected NG2-DsRed mice. B. Quantification the number of NG2 + cells(/mm2) in the injection region and para-injection region of the vehicle (2%DMSO) (n = 6) and Aβo-injected (n = 6) groups. C. Quantification of the number of PDGFRβ + cells(/mm2) in the injection region of vehicle (2%DMSO) (n = 6) and Aβo-injected (n = 6) groups. All the data were presented as mean ± SEM and analyzed using an unpaired t-test. ** p < 0.01 and **** p < 0.0001 [file 13195_2024_1423_MOESM2_ESM.tif]

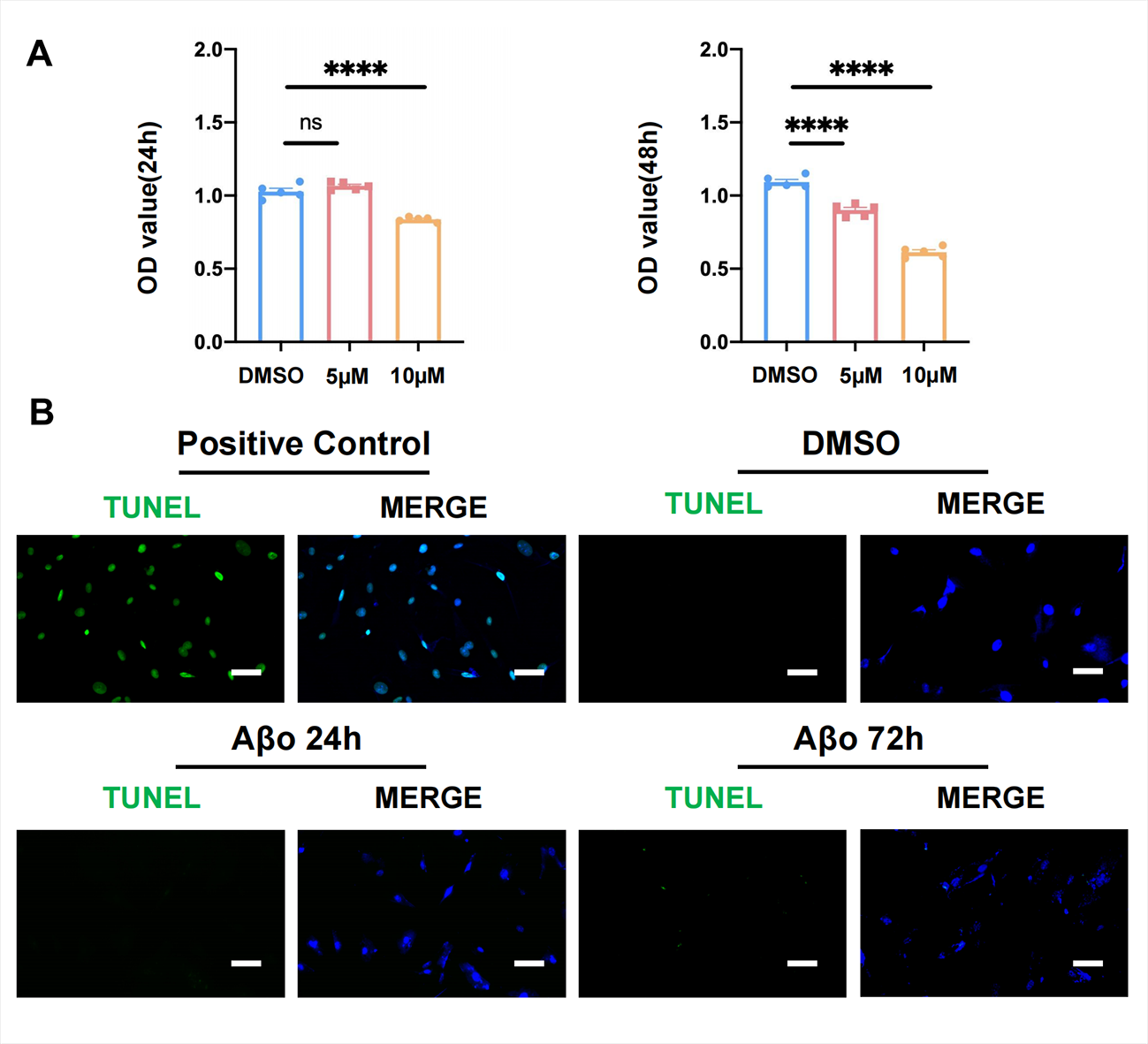

Supplement: Supplementary file 3 — Additional file 3: Figure S3. The viability of primary pericytes decreased after Aβo incubation and rare apoptosis was found in vitro. A. The OD value (450 nm) of primary pericytes detected by CCK-8 assay after incubated in media containing different concentrations of Aβ oligomers (0, 5, and 10 μM) for 24 h and 72 h. B. Representative images of tunel (green) and DAPI (blue) in primary pericytes incubated with Aβo for 24 h and 72 h. Scale bar: 50 μm. All the data were presented as mean ± SEM. **** p < 0.0001 [file 13195_2024_1423_MOESM3_ESM.tif]
