# Supplementary material for: Amyloid β oligomer induces cerebral vasculopathy via pericyte-mediated endothelial dysfunction
Source: Alzheimers Res Ther. 2024 Mar 12;16:56. doi: 10.1186/s13195-024-01423-w (PMC10935813; doi:10.1186/s13195-024-01423-w)
Supplement: Supplementary file 4 — Additional file 4: Supplementary Table 1. List of primers used in RNA analyses. [file 13195_2024_1423_MOESM4_ESM.docx]

**Supplementary Tables**

**Supplementary Table 1. List of primers used in RNA analyses.**

| Genes | primers | Sequence (5′–3′) |
| --- | --- | --- |
| *IL-1β* | Forward Primer | GCAACTGTTCCTGAACTCAACT |
|  | Reverse Primer | ATCTTTTGGGGTCCGTCAACT |
| *IL10* | Forward Primer | GCTCTTACTGACTGGCATGAG |
|  | Reverse Primer | CGCAGCTCTAGGAGCATGTG |
| *IL6* | Forward Primer | TAGTCCTTCCTACCCCAATTTCC |
|  | Reverse Primer | TTGGTCCTTAGCCACTCCTTC |
| *TNF-α* | Forward Primer | CCCTCACACTCAGATCATCTTCT |
|  | Reverse Primer | GCTACGACGTGGGCTACAG |
| *CXCL10* | Forward Primer | CCAAGTGCTGCCGTCATTTTC |
|  | Reverse Primer | GGCTCGCAGGGATGATTTCAA |
| *CXCL1* | Forward Primer | CTACTAGGAGCTGCGACACG |
|  | Reverse Primer | AAGCCACTGGGATTCGTGAG |
| *CCL2* | Forward Primer | CCAATGAGTAGGCTGGAGAGC |
|  | Reverse Primer | TCTCATTTGGTTCCGATCCAGG |
| *CCL5* | Forward Primer | GCTGCTTTGCCTACCTCTCC |
|  | Reverse Primer | TCGAGTGACAAACACGACTGC |
| *TGFβ* | Forward Primer | CTCCCGTGGCTTCTAGTGC |
|  | Reverse Primer | GCCTTAGTTTGGACAGGATCTG |
| *Cyclin D1* | Forward Primer | GCGTACCCTGACACCAATCTC |
|  | Reverse Primer | CTCCTCTTCGCACTTCTGCTC |
| *DAD1* | Forward Primer | TGAAGTTGCTGGACGCCTATC |
|  | Reverse Primer | AAGCCAGAGAGGAACGAGTTG |
| *Integrin b4* | Forward Primer | GCAGACGAAGTTCCGACAG |
|  | Reverse Primer | GGCCACCTTCAGTTCATGGA |
| *Integrin a5* | Forward Primer | CCGTGGACTTCTTCGAGCC |
|  | Reverse Primer | CTGTTGAATCAAACTCAATGGGC |
| *ACTIN* | Forward Primer | GACGGCCAGGTCATCACTATTG |
|  | Reverse Primer | CCACAGGATTCCATACCCAAGA |
